# Cytoplasmic transport and nuclear import of plasmid DNA

**DOI:** 10.1042/BSR20160616

**Published:** 2017-11-29

**Authors:** Haiqing Bai, Gillian M. Schiralli Lester, Laura C. Petishnok, David A. Dean

**Affiliations:** Division of Neonatology, Department of Pediatrics, University of Rochester, 601 Elmwood Avenue, Box 850, Rochester, NY 14642, U.S.A.

**Keywords:** cytoskeleton, cell nucleus, gene therapy, intracellular transport, microtubule, nuclear protein transport

## Abstract

Productive transfection and gene transfer require not simply the entry of DNA into cells and subsequent transcription from an appropriate promoter, but also a number of intracellular events that allow the DNA to move from the extracellular surface of the cell into and through the cytoplasm, and ultimately across the nuclear envelope and into the nucleus before any transcription can initiate. Immediately upon entry into the cytoplasm, naked DNA, either delivered through physical techniques or after disassembly of DNA–carrier complexes, associates with a large number of cellular proteins that mediate subsequent interactions with the microtubule network for movement toward the microtubule organizing center and the nuclear envelope. Plasmids then enter the nucleus either upon the mitotic disassembly of the nuclear envelope or through nuclear pore complexes in the absence of cell division, using a different set of proteins. This review will discuss our current understanding of these pathways used by naked DNA during the transfection process. While much has been elucidated on these processes, much remains to be discerned, but with the development of a number of model systems and approaches, great progress is being made.

## Introduction

Productive transfection and gene transfer require extracellular DNA to cross the plasma membrane, traffic through the cytoplasm, enter the nucleus, be transcribed, and have the resulting mRNA be exported into the cytoplasm for translation, and the ultimate modification and localization of the expressed protein. While most development of transfection reagents and strategies has focussed largely on carriers for plasma membrane targetting and entry and the choice of the ‘best’ promoter, these other steps of intracellular trafficking are crucially important for successful gene transfer.

## Formation of host protein–DNA complexes following internalization

Once DNA has been released successfully into the cytoplasm, it must traffic to the nucleus in order for gene expression to occur. This translocation across the cytoplasm represents a significant barrier to gene delivery. First, the cytoplasm contains nucleases that will degrade free DNA. Studies have demonstrated that plasmid DNA is degraded in the cytoplasm of HeLa and COS cells with a half-life of 50–90 min [1]. This poses a large problem for delivery of naked DNA and DNA–carrier complexes that are believed to dissociate prior to nuclear entry. However, the terms ‘naked DNA’ or ‘free’ DNA may not actually describe the state of DNA once released into the cytoplasm. Rather, any ‘naked’ DNA is rapidly complexed with a number of host cellular proteins to form a protein–DNA complex that mediates subsequent interactions with intercellular pathways, condenses the plasmid to reduce its effective size, and shields the DNA from rapid degradation.

**Figure 1:**
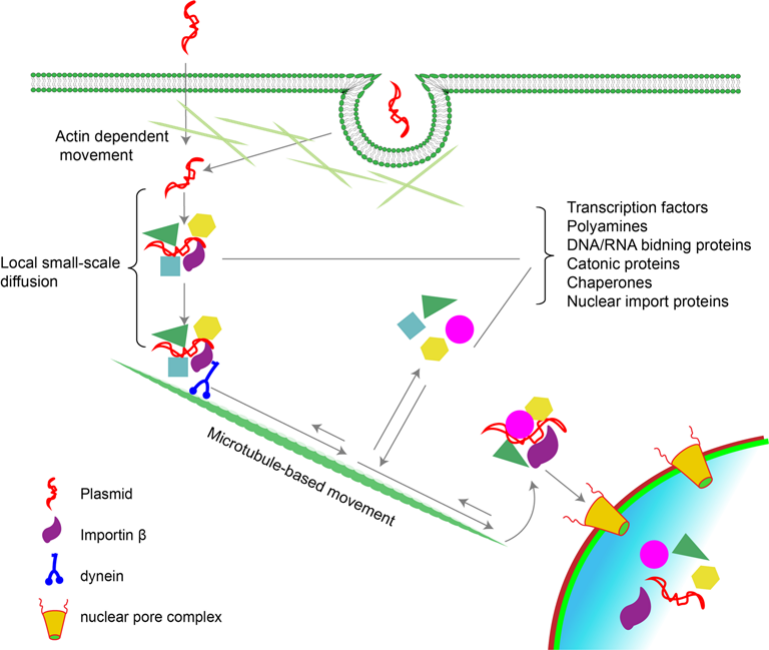
Intracellular trafficking of plasmids Most plasmids enter the cell by either endocytosis and/or direct entry into the cytosol at which point they must traverse the cortical actin layer, perhaps using actin-based movement [25,119]. Once free in the cytoplasm, plasmids are rapidly complexed by a number of DNA-binding proteins in the cytoplasm which in turn bind to other proteins to form large protein–DNA complexes [2]. Transcription factors bound to the DNA interact with importin β and other proteins that link the complex to dynein and kinesin for movement along microtubules toward the nucleus [19]. During this process, the DNA–protein complexes appear to be dynamic, with various proteins coming on and off the DNA at different times, perhaps to mediate different processes. Nuclear entry is then mediated by importin β in a sequence- and importin-dependent manner through the nuclear pore complex (NPC) in non-dividing cells or independent of importins and any DNA sequence requirement during mitosis and the associated dissolution of the nuclear envelope.

When naked DNA is delivered to adherent cells by electroporation and isolated as early as 15 min later, significant numbers of proteins can be found associated with the plasmid [2–4]. In the case of the GFP expression vector pEGFPN1 (Clontech), over 600 proteins are found to associate with the plasmid in each of three independent experiments [2]. While many of these proteins bind non-specifically to DNA or associate weakly by simple electrostatic interactions, a large number of proteins interact specifically with plasmids that productively traffic through the cell. Analysis of protein–DNA complexes over time in transfected cells also show that these complexes are dynamic with certain proteins remaining bound to the DNA for at least 4 h and others that bind early but come off at later times or those that come on at later times only [2]. Thus, it is likely that many of these complexes serve multiple roles in cytoplasmic movement along the cytoskeleton, protection from cytoplasmic nucleases, and nuclear localization (Figure 1).

## Cytoplasmic movement

The cytoplasm is a viscous environment crowded with molecules, which results in decreased mobility of macromolecules [5–7]. Spot-photobleaching and other studies have demonstrated that small solutes can diffuse freely and rapidly in the cytoplasm and the nucleus [5,8]. However, similar studies looking at the movement of labeled DNA fragments have shown that while small DNA can diffuse, those larger than 2000 bp were effectively unable to diffuse to any degree in the cytoplasm in any reasonable physiological time frame [6]. This is due to the existence of the cytoskeleton and the large numbers of actin filaments, microtubules, and intermediate filaments that form a highly cross-linked gel-sol [9–13]. Because of this system of networks, if DNA is released from an endosome at a site distant from the nucleus, the DNA cannot simply diffuse toward its desired location. This has been demonstrated in the case of liposome transfections where some DNA is left free in the cytoplasm and never reaches the nucleus [14,15]. Although it has been shown that lipoplex-containing endosomes themselves traffic toward the nucleus and the interior of the cell, there is still quite a lot of distance for the free DNA following endosomal release to move before it reaches the nucleus.

Since diffusion cannot account for the movement of plasmids to the nucleus, the only alternative is directed active transport (Figure 1). Indeed, we and others have shown that DNA in the cytoplasm utilizes the microtubule network and the molecular motor, dynein [16–18] for its trafficking to the nucleus. Early studies pointed to microtubules as being involved in the directed movement of plasmids based on the fact that upon microinjection into the cytoplasm of TC7 cells, GFP-expressing plasmids were able to drive high level GFP expression in non-dividing cells when untreated but showed greatly reduced GFP expression upon treatment with nocodazole, a microtubule disrupter [17]. By contrast, treatment with drugs that affected actin dynamics had no effect on transgene expression [17]. Further, co-injection of DNA with inhibitory antibodies against dynein also reduced subsequent gene expression, suggesting that without either intact microtubules or nuclear-directed dynein, cytoplasmic plasmids were unable to make their way to the nucleus for gene expression [17]. *In vitro* binding assays confirmed that plasmids do indeed interact with microtubules through cytoplasmic adapter proteins, including dynein and transcription factors [17,19]. Interestingly, when different plasmids were analyzed for their ability to interact with microtubules in this binding assay, it was found that binding sites for the transcription factorcAMP response element binding protein (CREB) were required for the interaction; when plasmids lacking CREB sites were used, no interactions were detected, suggesting some degree of DNA sequence specificity in the interaction and for movement [19].

These studies were confirmed by following individual fluorescently labeled plasmids in microinjected cells using particle tracking [19]. When plasmids carrying CREB-binding sites, which are fortuitously present in multiple copies in the cytomegalovirus (CMV) immediate early promoter, were cytoplasmically microinjected, directed movement was observed with initial velocities of 150 nm/s and up to 380 nm/s, indicative of directed, dynein-driven movement of proteins and organelles along microtubules [20–23]. By contrast, when a plasmid lacking CREB sites was followed, the observed velocities were less than 50 nm/s [24], a rate of movement indicative of random Brownian motion or limited diffusion. Similar rates and directionality of plasmid movement have also been seen following electroporation-mediated delivery of plasmids in cultured cells, largely confirming our earlier findings [25]. This study also showed that at early times after electroporation, the actin network and associated motors may also play a role in DNA movement from the periphery of the cell to the microtubules themselves since treatment of cells with drugs that affect actin dynamics reduced plasmid velocities and displacement of the particles but did not greatly affect total plasmid movement [25]. Proteomic studies from our laboratory have found that several actin-based motors (myosin 1B, 1C, and 9) are found in protein–plasmid complexes at early times after electroporation (15 min) along with a number of different microtubule-based motors [2]. This supports a possible role for actin-based movement of DNA particles, at least at times between entry of the DNA into the cytosol and its binding to microtubules (Figure 1). However, since the actin network and its associated motors are known to play critical roles in the internalization of endosomes and their subsequent intracellular movement, it is also possible that the effects of actin filament disruption could be due to movement of vesicles, rather than the cytoplasmic DNA itself.

## Directed trafficking of plasmids in the cytoplasm

Since DNA has not been shown to bind directly to dynein, the mechanism of this interaction was investigated and was found to involve a multiprotein complex that bridges the DNA to microtubules and their associated motors. *In vitro* binding assays revealed that plasmid DNA can interact with microtubules only in the presence of cellular extracts [17]. When plasmids carrying different eukaryotic promoters were tested for their ability to interact with microtubules in this assay, it was found that while plasmids carrying either the CMV or cauliflower mosaic virus 35S promoter bound efficiently to the microtubules in the presence of cell extract, plasmids carrying either no promoter or a number of other different RNA polymerase II promoters failed to do so [19]. Analysis of the transcription factor binding sites present in these DNAs revealed that binding sites for the transcription factor CREB mediated this interaction. An *in vivo* role for this binding was demonstrated by pull-down assays in transfected cells. Plasmids containing CREB binding sites co-precipitated with CREB as early as 15 min after electroporation of adherent cells, but plasmids without CREB-binding sites showed no such interaction [19].

The functional consequence of this interaction was shown by investigating the initial velocities, through particle tracking, of microinjected plasmids with or without CREB-binding sites [19]. A bacterial plasmid containing no eukaryotic promoter showed very limited movement following microinjection, indicative of limited diffusive motion. By contrast, plasmids carrying CREB sites in the CMV promoter showed rapid and directional movement consistent with microtubule-based trafficking. When another plasmid carrying SV40 enhancer but no CMV promoter or CREB-binding sites was injected, the plasmids also showed directional active transport, although at lower rates than seen with the CREB site containing plasmids. When CREB was depleted from cells using siRNA, no change in the initial velocities were seen for the bacterial plasmid or the SV40 enhancer only plasmid, but a statistically significant drop in velocity was detected for plasmids carrying CREB-binding sites, suggesting that the binding of this protein has functional consequences for cytoskeletal movement of the DNA.

In studies on the composition of the protein–DNA complexes that traffic through the cytoplasm, a number of other proteins have been found to associate with DNA and also may play a role in microtubule-based movement [2]. Moreover, most of these proteins are much more abundantly associated (from 3- to 250-fold) with trafficking plasmids compared with those plasmids that displayed no directed movement, based on their sequence makeup. Several of these are known microtubule-associated proteins (MAPs), including MAP1B and MAP4, a microtubule-actin cross-linking factor, and several known motor proteins, including kinesin-1, kinesin 5B, and dynein-1. Additional proteins in the trafficking DNA complex included the nuclear localization signal receptor proteins importin β1, importin 4, importin 7, importin α1, and importin α2, as well as numerous DNA-binding proteins and chaperones. While not all have been tested for their roles in cytoplasmic DNA movement, several of these proteins have been shown to play an active role in cytoskeletal trafficking, since their depletion by siRNA treatment, greatly inhibited the ability of plasmids to move in the cytoplasm. For example, siRNA silencing of importin β1 blocked all directed movement of injected plasmids and reduced the movement of the plasmid to less than 50 nm/s, whereas depletion of importin 7 or importin α1 had no effect on plasmid velocities in the cytoplasm [2]. Another member of the importin family was also found in the trafficking DNA complexes: exportin, also called Crm1. This protein does not bind to nuclear localization signals (NLSs) to facilitate nuclear import, but rather is the major nuclear export signal (NES) receptor that drives nuclear export of proteins. As in the case of importin β1, inhibition of NES binding by exportin using the drug leptomycin B abolished the ability of plasmids to move along microtubules in the cytoplasm, suggesting that this protein, perhaps in association with key exportin cargo proteins, is actively involved in cytoskeletal trafficking of DNA. This is not unprecedented, since exportin has also been shown to be needed for movement of adenoviral particles from the microtubule organizing center to nuclear pores in the nuclear envelope, although not movement on the microtubules, *per se* [26].

Another recent study has implicated several kinesin family members as playing a role in the movement of small dsDNA fragments along microtubules [27]. When 32-bp oligonucleotides labeled with quantum dots were internalized by cells, a fraction of them showed directed movement consistent with active transport, but while roughly a half were directed toward the nucleus, the other half was directed toward the cell periphery. When biotinylated oligos were used for pull-downs, three plus- (periphery) directed kinesins (KIF1C, KIF4A, and KIF14), the minus-directed C-terminal kinesin KIFC1 and the cytoplasmic dynein-1 were found to be amongst the proteins in the DNA complexes [27]. FRET experiments were suggestive of a direct interaction between KIFC1 and the DNA, although indirect interactions could not be ruled out. Given that KIFC1 drives movement toward the nucleus, it is possible that this motor may play a role in plasmid movement to the nucleus as well.

## Modulation of microtubule-based DNA movement

The cytoskeleton is a highly dynamic network that is influenced by external and internal forces. A host of post-translational modifications to actin or tubulin monomers and to the filaments and microtubules themselves can have profound effects on loading and unloading of cargoes and their movement once bound to these networks. One such modification is the hyperacetylation of microtubules which leads to increased recruitment of both kinesin and dynein motors and their bound cargo [28–30]. Several viruses, including HIV, some herpes viruses, and circoviruses, also show greater cytoplasmic trafficking and enhanced infection when microtubules are hyperacetylated [30–34]. We found that exposure of cultured cells to mild levels of cyclic stretch leads to the hyperacetylation of microtubules and increased binding of plasmid DNA to microtubules in the cell, more rapid movement into the nucleus along the microtubules, and greater transfection efficiency [35,36]. This effect was mediated through the inhibition of histone deacetylase 6 (HDAC6), a cytoplasmic deacetylase whose major target is tubulin; silencing of HDAC6 by siRNA or inhibition of HDAC6 by drugs lead to the same result, but overexpression of HDAC6 could alleviate the enhanced DNA binding, cytoplasmic trafficking, and increased transfection efficiency seen with cyclic stretch. When particle tracking was used to quantitate the effects of microtubule hyperacetylation on plasmid movement in cells, it was found that increased tubulin acetylation not only leads to increased numbers of plasmids loading on to the microtubules, but also leads to greater processivity of DNA transport along the microtubules [35]. Similar increased gene transfer and expression with cyclic stretch or HDAC6 inhibition were seen in the lungs of living mice following brief, high tidal volume ventilation or treatment with HDAC6 inhibitors when plasmid DNA was delivered using transthoracic electroporation [37,38]. These results demonstrate that cytoplasmic trafficking is important for gene delivery in both cells and tissues.

## Nuclear envelope as a barrier to transfection

For years, the nuclear envelope has been proposed to be one of the most substantial barriers for DNA delivery in cells and tissues. Capecchi [39] carried out an elegant set of experiments in 1980 in which he microinjected plasmids into either the nuclear or cytoplasmic compartment of non-dividing cells and assayed their ability to express their gene product. When pBR322-based plasmids were injected into the cytoplasm, 100% of the injected cells showed no gene expression, but when the plasmids were injected into the nucleus, 50–100% of cells showed gene expression [39]. Over the next 20 years, several other groups demonstrated similar findings in multiple cell types and even *Xenopus* oocytes, showing that the nuclear envelope was a major impediment to effective gene transfer in the absence of cell division [40–43].

Despite these studies, it is clear that transfected DNA does reach the nucleus, although the amount that does may be small. It has been estimated that between 2000 and 10000 plasmids are delivered per cell following lipofection, but that only between 20 and 1000 are detected in the nucleus by 24–36 h following DNA addition [44–46]. Even in actively dividing cells, microinjection into the cytoplasm takes between 30- and 100-times more plasmid compared with injection into the nucleus to give equivalent levels of gene expression [47,48]. These studies and others clearly illustrate both that cytoplasmic trafficking is inefficient and that nuclear entry can be a significant issue.

## Nuclear localization in mitotic cells

In the absence of cell division, the intact nuclear envelope remains largely impermeable to plasmids and plasmid–carrier complexes. However, during mitosis, the nuclear envelope breaks down, allowing cytoplasmic plasmids near the nucleus access to the nuclear space. During mitosis, chromosomes are highly condensed by a number of resident nuclear proteins that interact largely though non-specific interactions with DNA. Many of these proteins, most notably histones, become dephosphorylated by several protein phosphatases that are up-regulated during mitosis. This dephosphorylation leads to the recruitment of sets of proteins that facilitate interaction with nuclear envelope proteins present in the disassembled envelope (as sheets and vesicles) for engulfment of the chromatin and subsequent reformation and sealing of the nuclear envelope [49]. Since plasmids become rapidly chromatinized once internalized [50–53], they too may provide a scaffold on which the nuclear envelope is deposited and reformed, as has been shown *in vitro* [54,55]. More efficient nuclear localization and transfection efficiency in dividing cells also has been noted in transfections using a number of different carriers, including liposomes, polyethyleneimine, and nanoparticles [56–59]. For example, one study demonstrated that actively dividing cells were ten times more likely to express the transferred gene product than cells that had not divided [60]. In another study that looked at the redistribution of transfected plasmids following subsequent mitosis, it was shown that even plasmids that cannot enter the non-dividing nucleus (see below) partition relatively equally to daughter nuclei, suggesting that the plasmids are trapped in the reforming nuclei and not transported in after nuclear formation [61].

## Nuclear localization in the absence of cell division

Although nuclear localization of plasmids is much more efficient in dividing cells and leads to much higher transfection efficiencies using almost all the techniques, DNA is able to enter the nuclei of non-dividing cells, albeit to a much less extent. Indeed, if this were not the case, there would be effectively no gene transfer to any tissue in living animals or humans since the majority of cells in tissues are either terminally differentiated or divide with doubling times of weeks to months. In the absence of mitosis and the subsequent breakdown of the nuclear envelope, the only way for proteins and protein–DNA complexes to enter the nucleus is through nuclear pore complexes (NPCs). The NPC is an aqueous channel in the nuclear envelope through which proteins, ribonucleoproteins, and all macromolecules in the cell can traffic between the cytoplasm and nucleus [62]. The pores are large (~125 MDa) multiprotein complexes that are composed of more than 100 distinct proteins present in multiple copies [63]. Transport through the NPC occurs either by signal-independent diffusion in the case of small molecules such as nucleotides, ions, and solutes, or by signal-mediated facilitated diffusion.

Nuclear entry of plasmids in the absence of cell division was first directly observed after plasmids were microinjected into the cytoplasm of primary myotubes [64]. Wolff and colleagues showed that the biotin-labeled plasmids were able to enter nuclei of myotubes and to direct gene expression [64]. This nuclear import was a facilitated process that was dose and energy dependent and was inhibited by agents that block transport through the NPC. This suggested either that karyophilic proteins containing NLSs enabled the injected plasmids to enter nucleus or that the DNA itself may have an intrinsic signal for nuclear pore recognition and nuclear entry. They also demonstrated that greater levels of gene expression (hence trafficking) were obtained when the plasmids were injected near as opposed to far away from the nuclei [64]. Using a similar approach, our laboratory demonstrated that plasmids do indeed traffic into the nucleus through the NPC by localizing the injected DNA by *in situ* hybridization [65] or directly using plasmids labeled with fluorescent peptide nucleic acid (PNA) clamps [66] in a process that is inhibited by agents that block signal-mediated transport through the NPC. More recently, experiments have shown that this import is size dependent as well, with smaller plasmids or those compacted with protamine being able to localize to the nucleus and express their products faster than larger ones [67].

In case of proteins, the signals needed for transport are either the NLS for nuclear import or the NES for nuclear export. Although a number of microinjection studies have shown that proteins less than 60 kDa in size have the ability to diffuse through the NPC for nuclear entry [68], even small nuclear proteins contain NLSs that are required for nuclear localization. RNAs, including mRNA, snRNAs, miRNAs, and tRNAs, all exist as ribonucleoprotein complexes that require both RNA and protein signals for their export or import into the nucleus. In all cases, the signals needed for nuclear import or export are recognized by a class of receptors termed the karyopherins [69–71]. These fall into two general classes: importins for nuclear import and exportins for nuclear export. In the case of the importins, the NLS on a cargo protein is recognized either directly by a β subunit (of which there are 11 in humans) with β1 being the prototype, or by an α subunit (7 isoforms in humans) that then binds to β1 for translocation across the NPC. Similar to importin α, other factors can recognize motifs on RNA, such as the trimethylguanosine (m3G) cap structure of the U snRNA which is recognized by snurportin 1, and interact with importin β subunits for complex import. In the case of nuclear export, one of the seven exportins interacts directly with the NES to facilitate export. The ‘directionality’ of transport is controlled by the small Ras-like GTPase Ran in its bound GTP or GDP state. GTP exchange factors reside in the nucleus while GTPase activating proteins are maintained in the cytoplasm, leading to a gradient of Ran-GTP in the nucleus and Ran-GDP in the cytoplasm. Assembly of export complexes (NES cargo bound to exportin) is driven by association with Ran-GTP in the nucleus while disassembly of import complexes (NLS cargo bound to importin β) is promoted by binding to Ran-GTP in the nucleus, thus leading to this directionality.

**Figure 2:**
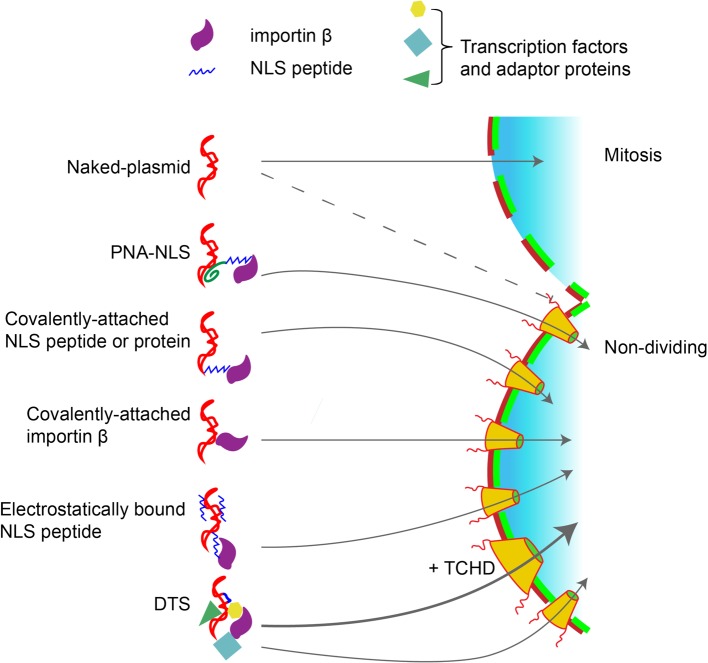
Strategies to increase nuclear targetting of plasmids A number of different approaches have been used to improve the nuclear import of plasmids, all of which center around attaching NLS peptides, or NLS proteins, or other importin interacting peptides to the DNA. This can be also done by increasing the functional diameter of the NPC itself by enhancing non-selective gating of the pore with the drug TCHD [120,121].

**Figure 3:**
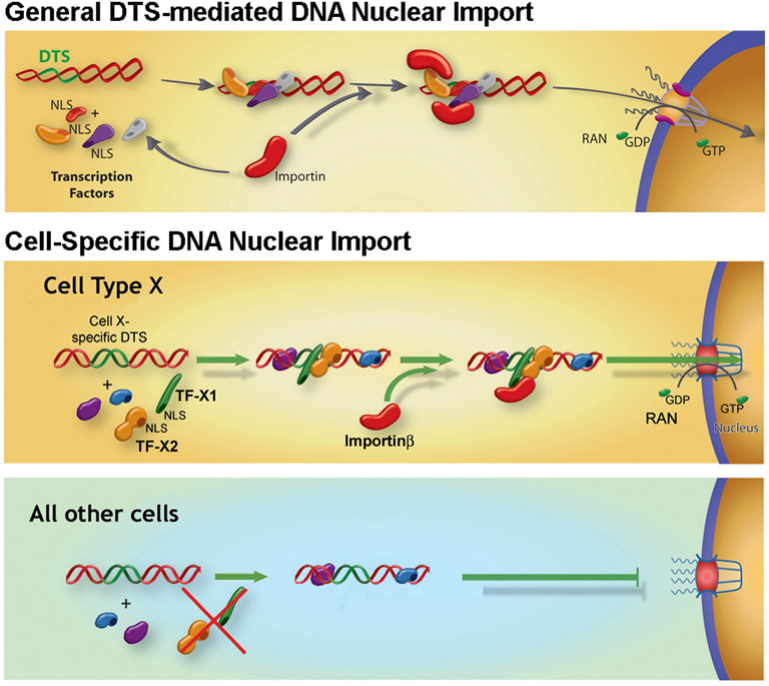
Model for general and cell-specific DNA nuclear import in non-dividing cells If plasmids containing sequences that act as scaffolds for transcription factors and other DNA-binding proteins (termed DTS or DNA nuclear targetting sequences) are deposited into the cytoplasm during transfection, they can form complexes with these proteins, thereby attaching NLSs to the DNA. Some, but not all, of these NLSs may be in a conformation able to interact with importins for transport of the DNA–protein complex into the nucleus through nuclear pores. In the case of general DTSs, the transcription factors that interact with the DTS are ubiquitously expressed and thus allow DNA nuclear import in all the cell types. By contrast, cell-specific DTSs bind to a group of cell-specific transcription factors as well as some general transcription factors to form import competent complexes in corresponding cell types (the cell type X DTS binds cell type X-specific transcription factors in cell type X). In other cell types, the specific transcription factors are absent and consequently, plasmids fail to be imported into the nuclei for expression.

Since DNA itself is not a protein, it contains no intrinsic NLS, and thus cannot simply use the importin machinery directly for its nuclear import. The simplest model for DNA movement through the NPC would be for the DNA to attract and associate with NLSs. This strategy has been used in many iterations in attempts to increase the nuclear targetting of plasmids during transfections. NLS peptides, either free or as conjugates, exogenous NLS-containing proteins, importin domains, ligands for nuclear hormone receptors, and other approaches have all been tested to increase DNA nuclear import, all to varying degrees of success (Figure 2) [72,73]. This NLS attachment can also be achieved by DNA interacting with endogenous DNA binding proteins, such as transcription factors, bound for the nucleus while both the proteins and the DNA are in the cytoplasm (Figure 3). To function, transcription factors consist of at least three domains or activities: an NLS for nuclear entry, a DNA-binding domain to interact with specific DNA sequence(s), and a domain that regulates transcription in some manner. If transcription factors encounter DNA in the cytoplasm, they can (and do) form protein–DNA complexes that may have one or more exposed NLSs that can in turn be recognized by the importins to carry the entire complex through the NPC into the nucleus. If this model is correct, most plasmids should be able to enter the nucleus in the absence of cell division, since many DNA-binding proteins and factors involved in transcription in the cell have the ability to bind to DNA either specifically or non-specifically and should create these transport-competent plasmid complexes. However, this does not appear to be the case.

## DNA nuclear targetting sequences

The first suggestion that plasmids may contain specific sequences that drive nuclear import better than others came from microinjection and injection studies in the 1980s. SV40 is a DNA tumor virus that infects non-dividing cells and expresses large T-antigen, a protein with transforming activity, which can lead to transformation and tumorigenesis. In studies to understand the mechanisms of expression of these viral genes, various eukaryotic control regions of the SV40 virus were cloned into plasmids and evaluated for expression. When plasmids were injected directly into the nucleus of dividing cells, those containing the SV40 enhancer showed only modestly increased expression compared with those injected into the cytoplasm. However, when the enhancer sequence was not present in a plasmid, injection into the cytoplasm showed 30-fold lower levels of expression compared with delivery directly into the nucleus [40]. These studies suggested that the SV40 enhancer may have some type of ‘helper’ activity to aid in viral infection and expression, although the mechanisms were not elucidated or proposed. In later microinjection and *in situ* hybridization studies to investigate movement of the DNA itself, it was found that plasmids containing the entire SV40 genome (5243 bp) or just 50 bp of the SV40 enhancer were actually able to enter the nuclei of growth-arrested cells prior to cell division, while those lacking the enhancer remained in the cytoplasm until the cells divided [47,65]. This sequence specificity of plasmid nuclear import has been seen in all cultured cells tested to date, including established cell lines and primary cells from multiple species and multiple cell and tissue lineages, suggesting that this is a general phenomenon.

The SV40 enhancer is a 72-bp sequence that is present in two tandem copies in the SV40 genome. It contains binding sites for at least ten different transcription factors, AP1, AP2, AP3, NF-κB, Oct-1, TEF-I, and TEF-II, all of which are general factors that are ubiquitously expressed in mammalian (and most eukaryotic) cells [74–76]. The ubiquity of these transcription factors could explain why sequence-specific plasmid nuclear import is seen in all cell types tested so far. The need for this sequence for effective plasmid nuclear import also fits nicely with the model for DNA nuclear import, since the enhancer provides a scaffold for transcription factor binding to ‘coat’ the plasmid or at least one region of it, with NLS-containing proteins. Sequences such as the SV40 enhancer have been termed DNA nuclear targetting sequences (DTSs) [65].

Based on the activity of the SV40 enhancer and the model for plasmid nuclear import in which binding of transcription factors to the DNA can facilitate access to the nucleus, a likely possibility is that any eukaryotic promoter or transcription factor binding site placed into a plasmid could elicit the same function. However, this does not seem to be the case. Several strong viral promoters have been tested for their nuclear targetting ability, but none of them showed similar ‘DTS’ activity. The immediate early promoter and enhancer from CMV, the Rous sarcoma virus long terminal repeat (LTR), the Moloney murine leukemia virus LTR, and the herpes simplex virus thymidylate kinase (TK) promoter are unable to mediate plasmid nuclear import in non-dividing cells [40,47,77]. One possible reason is that these other promoters do not contain binding sites for a specific transcription factor that may be responsible for transport activity. Alternatively, a specific combination of factors is needed that is also not built on these other sequences. These two possibilities suggest that not all transcription factors are capable of transporting bound DNA into the nucleus.

The normal ‘life cycle’ of a transcription factor is to be synthesized in the cytoplasm and then be transported into the nucleus using its NLS (either immediately after translation or upon signal-mediated stimulation such as tumor necrosis factor-α (TNF-α) stimulation of NF-κB). Only after the transcription factor has entered the nucleus does it encounter the DNA and bind to it. Many transcription factors, including many basic helix–loop–helix (bHLH) and zinc-finger proteins, contain NLSs and DNA-binding domains that are coincident and overlapping. For example, the NLS of the fos and jun proteins that make up the AP1 transcription factor are encoded within the DNA-binding domains of this helix–loop–helix factor. By contrast, other transcription factors, such as NF-κB, have spatially distinct domains for their NLS and DNA-binding activities so that both can function simultaneously to facilitate the cytoplasmic trafficking and nuclear import of plasmids from the cytoplasm. Indeed, NF-κB has been shown to mediate the nuclear import of plasmids and the presence of tandem NF-κB-binding sites within a plasmid acts as a general DTS that is functional in all cell types [16,78–81].

To date, several general DTSs have been identified, largely on the basis of microinjection and subcellular localization studies, although several have been identified based on their ability to increase gene expression following microinjection or transfection (Table 1). Apart from the ~50-bp SV40 DTS that contains binding sites for multiple transcription factors and the NF-κB DTS composed of tandem NF-κB-binding sties on a plasmid, binding sites for the glucocorticoid receptor have also been reported to have similar DNA import activity [82]. This glucocorticoid receptor DTS activity was shown to improve nuclear localization and gene expression in confluent cells as well as in the lungs of mice following tail vein injection of cationic liposome–DNA complexes or aerosol delivery of polyethyleneimine–DNA complexes [82]. These studies are based on the earlier findings that complexation of plasmids with the glucocorticoid receptor by conjugating dexamethasone (a glucocorticoid receptor agonist) to the DNA resulted in greater DNA nuclear localization and gene expression [83,84].

**Table 1:** Examples of DTS

DTS	Cell specificity	References
SV40 enhancer	General	[65]
Glucocorticoid receptor binding sites	General	[82]
NF-κB-binding sites	General; inducible by TNF-α	[78]
Hypoxia response elements	General; inducible by hypoxia	[96]
Smooth muscle γ actin (SMGA) promoter	Smooth muscle cells	[77]
Flk-1 promoter	Endothelial cells	[91]
hcolA1 promoter	Osteoblasts	[92]
Surfactant protein C (SPC) promoter	Type II alveolar epithelial cells	[94]
Sox2 regulatory region 2	Embryonic stem (ES) cells	[95]
T1α promoter	Type I alveolar epithelial cells	[93]

One caveat to the use of the DTS strategy is that the DTS only works as anticipated in cells that are not dividing. When the cell goes through mitosis, the nuclear envelope breaks down and reassembles after cell division. Thus, any plasmid that is in the cytoplasm during mitosis is no longer prevented from entering the nuclear space and when cell division is finished, plasmids wind up being trapped within the new nuclear space. In this case, a DTS is not required for nuclear uptake [61]. Further, while the dependence on the DTS for plasmid nuclear import appears almost absolute in cultured cells, a number of studies have shown that in many tissues, especially skeletal muscle, robust gene transfer, and expression can be obtained using plasmids lacking any nuclear import sequence. It is likely that when the cytoplasm becomes filled with large concentrations of plasmids, at least some of the plasmids can make their way to the nuclear envelope and be imported into the nucleus independent of any DTS. Indeed, when linear DNA is brought close enough to the nuclear envelope using laser tweezers, it is pulled in [85]. It has also been shown that when DTS-lacking plasmids are delivered to the cytoplasm of a mouse myotube *in vivo*, no gene expression is observed until 1000000 plasmids or more are injected, suggesting that mass action could account for the nuclear localization [86].

Several studies aimed at evaluating the effects of the DTS on gene expression following transfection have reported seeing limited to no benefit from the DTS [87,88]. In several cases, gene expression was followed as the readout for nuclear entry, whereas the studies showing that a DTS (such as the SV40 DTS or tandem NF-κB sites) promoted nuclear import relied on direct imaging of plasmids following microinjection to isolate the step of nuclear import or by quantitating levels of nuclear DNA [47,65,79,81,89]. However, changes in gene expression can be affected at multiple points during the transfection process, from binding to the plasma membrane and internalization, compaction, release from carriers, nuclear import, and transcription itself. One elegant study from the Crommelin and Mastrobattista groups at Utrecht showed that no increased gene expression was seen from CMV promoter driven expression plasmids with the SV40 DTS in A431 and HeLa cells transfected by a number of different techniques [87]. The authors proposed that the use of the strong CMV promoter obscured or overcame any effects of preferential DNA nuclear import provided by the DTS and that any limitation in gene expression was at the level of post-transcriptional processing or translation, not pretranscriptional events. By contrast, they postulated that when a weak promoter is used, the transcription machinery would not be saturated, and any increase in nuclear delivery of the plasmids would lead to increased transcription and translation.

## Cell specific and regulated DNA nuclear entry

Based on the models of bound transcription factor mediated DNA nuclear import, it was postulated that cell-specific nuclear targetting sequences may exist [47]. It is possible that binding of cell-specific transcription factors to a plasmid could lead to cell-specific nuclear import in the cells in which these particular transcription factors are expressed (Figure 3). In order to identify such sequences, multiple cell-specific promoters were cloned into plasmids and tested for their ability to facilitate plasmid nuclear entry following microinjection in the specific cell types in which the promoters are transcriptionally active, but not in other cell types in which the promoters do not function. The first such cell-specific DTS identified was a 176-bp fragment of the smooth muscle specific smooth muscle γ actin (SMGA) promoter [77]. This fragment of the SMGA promoter was able to drive nuclear import of a plasmid in primary smooth muscle cells from multiple species, but did not cause nuclear import in other cell types including endothelial cells, fibroblasts, or epithelial cells [77,90]. Nuclear import of the SMGA DTS is mediated by two transcription factors that are preferentially coexpressed in smooth muscle, Nkx3.1/3.2 and serum response factor (SRF), both of which are necessary and sufficient for DNA nuclear uptake in these cells [77,90]. When the binding sites for these factors were mutated within the SMGA promoter, plasmids containing the mutant DTS remained in the cytoplasm of microinjected cells. Similarly, when Nkx3.1/3.2 and SRF were knocked down in smooth muscle cells through the use of siRNA, nuclear import of plasmids carrying the wild-type SMGA promoter was abolished, again showing that these factors are necessary for DNA nuclear import [90]. Sufficiency of these two transcription factors alone was shown by expressing the factors in bacteria, complexing the purified proteins with SMGA DTS plasmids prior to cytoplasmic microinjection, and obtaining nuclear import in non-smooth muscle cells that do not normally express these factors [90]. To date, at least five other cell-specific DTSs have been identified, including those active in endothelial cells [91], osteoblasts [92], type I alveolar epithelial cells [93], type 2 alveolar epithelial cells [94], and embryonic stem (ES) cells [95] (Table 1).

Using the same concept of coupling specific transcription factors and their binding sites for DNA nuclear import, an intriguing study from a group in Brazil demonstrated that binding sites for the hypoxia-inducible factor HIF-1α could function as a DTS in a controlled manner [96]. A293T cells were transfected with plasmids carrying either the 72-bp SV40 DTS or a 50-bp hypoxia-responsive element (HRE) using calcium phosphate and their nuclear plasmid content quantitated by PCR 4 h later. Under normoxic conditions, approximately twice as many SV40 DTS plasmids were present in the nuclei of transfected cells compared with the HRE plasmid, which was only slightly enriched in the nuclei of normoxic cells compared with a plasmid containing no DTS (20% more HRE plasmids in the nucleus compared with no DTS plasmid). When cells were grown under hypoxic conditions following transfection, the numbers of SV40 DTS plasmids in the nucleus dropped to the level of those seen with no DTS plasmid while the HRE plasmid showed a two-fold increase in nuclear plasmid.

## Effect of DTSs in animal models

DTS elements have also been shown to act in living animals as they do in cultured cells. In studies of vascular gene transfer, plasmids containing the SV40 DTS increased gene expression 40- to 200-fold after electroporation-mediated transfer into the mesenteric vessels of living rats [97]. Similar results were seen when plasmids expressing GFP or luciferase were quantitated. *In situ* hybridization verified that the increased reporter gene expression in rats electroporated with constructs containing the SV40 DTS compared with constructs lacking this sequence was a result of increased nuclear import and localization of the DTS plasmids [97]. As stated above, these DTS have benefit only in cells in the absence of cell division. When we denuded endothelial cells from the rat carotid by angioplasty and induced rapid smooth muscle and endothelial cell proliferation, no difference in the levels of gene expression were seen with plasmids carrying or lacking an SV40 DTS (Young and Dean, unpublished work). Similar effects of the SV40 DTS on increasing gene expression have also been reported in skeletal muscle using injection of DNA and electroporation for gene delivery, although these studies only followed expression levels and did not correlate nuclear plasmid levels with increased gene expression [98,99].

Cell-specific DTSs also have been shown to be cell selective in animals as they are in cultured cells. When the rat mesenteric vasculature was electroporated with CMV promoter driven GFP plasmids that contained either no DTS, the SV40 DTS or the SMGA DTS downstream of the *GFP* gene and evaluated 2 days later for gene expression, little to no expression was seen in vessels receiving the no DTS plasmids [100]. By contrast, those electroporated with the SV40 DTS plasmid showed robust expression throughout the neurovascular bundle (including smooth muscle cells, endothelial cells, fibroblasts, and adventitial cells). The SMGA plasmids also showed robust gene expression, but the expression was limited only to smooth muscle cells. When *in situ* hybridization was used to localize the delivered plasmids, the SV40 DTS plasmid could be seen in all cells within the vessel wall, but the SMGA DTS plasmid was only detected in smooth muscle cells and not in any other cell type, mimicking the expression results [100]. Taken together, these results demonstrated that the DTS enhanced nuclear localization of the transfected plasmids which in turn led to increased gene expression. Similar results have been seen in the lung for both type I- and type II alveolar epithelial cell specific DTSs: whereas the type II cell-specific surfactant protein C (SPC) DTS promoted nuclear import and gene expression disproportionately in type II cells, the type I cell-specific T1α DTS was restricted in its expression to type I alveolar epithelial cells, with over 80% of the gene expressing cells being type I cells [93,94]. Again, as in the dividing carotids following angioplasty, DTS function in the lung was also dependent on cell division. Although the majority of the cell-specific DTS activity was indeed in the corresponding target cells, some gene expression was detected in off-target cells. However, when animals were labeled with BrdU during the gene transfer process, off-target cells showing gene expression were also those that were BrdU positive, indicating that they had divided during the course of the experiment [94].

## Cofactors involved in DNA nuclear transport

The core proteins that serve as linkers between the DNA and the importin machinery are the specific transcription factors that interact with the various DTS. In the case of the SV40 DTS, these include a number of ubiquitously expressed mammalian transcription factors such as AP1, AP2, NF-κB, Oct1, TEF-1, and SP1, while in the case of the various cell-specific DTSs, cell-specific transcription factors are required that vary with the cell type and DTS involved. To dissect the machinery required for DNA nuclear import, a number of experimental systems have been used to identify other protein factors involved in this import. One of the most useful systems for reconstitution has been the digitonin permeabilized HeLa cell system established by Adam and colleagues [101]. Incubation of cells with relatively low concentrations of the detergent digitonin preferentially permeabilizes the plasma membrane, allowing the cytosolic contents of the cell to be removed. By adding back purified components of the nuclear import machinery (e.g. importin α, importin β, Ran etc.), the roles of specific proteins and importins can be determined. Using this system, we showed that the nuclear import of plasmids fluorescently labeled at a specific site with a PNA conjugated to fluorescein was able to accumulate in the nucleus of cells in the presence of complete cytoplasmic and nuclear extracts [66]. Import was detectable as early as 60 min after addition of DNA and saturated by 4 h. This import was energy dependent and could be blocked with either lectins or antibodies that bind to and block NLS-mediated movement through the NPC. When bacterially expressed and purified importin α, importin β, and Ran were added in lieu of cytoplasmic extracts, nuclear import of an NLS-containing protein was reconstituted, but that of plasmid DNA was not unless nuclear extracts were also supplied. Like nuclear import in microinjected cells, plasmid nuclear import in the permeabilized cells was sequence dependent. While plasmids carrying the SV40 DTS showed robust nuclear accumulation in the presence of importin α/β and Ran, plasmids without the SV40 sequence failed to accumulate [66].

This requirement for the soluble NLS import machinery for plasmid nuclear localization appears to be in contrast with the results of Wolff and colleagues who found that the nuclear import of fluorescently labeled linear DNA fragments was energy dependent and inhibited by wheat germ agglutinin but occurred optimally in the absence of cytoplasmic proteins [102]. One likely reason for this disparity is that the uniformly labeled, short, linear DNA used in the Wolff study is a very different substrate compared with plasmid labeled at a discrete site, and as such, may utilize different pathways for entry.

Proteomics has been used to identify the proteins that complex with plasmids either in solution or in cells during the transfection process [2,3,103]. In *in vitro* studies, cell extracts were subjected to affinity chromatography using supercoiled plasmids immobilized on to columns at a single, specified site through the use sepharose-conjugated PNAs [3,103]. Subsequent MS analysis identified a number of proteins that bound to plasmids able to enter the nuclei of non-dividing cells (i.e. containing the SV40 DTS) or those that did not (pBR322). In the Miller and colleagues [3] study, several proteins involved in NLS-mediated nuclear import were identified to bind to the SV40 DTS plasmid but not pBR322, including importin β1, importin 7, Ran, RanBP1, and RanBP2, as were numbers of transcription factors, DNA processing factors, chromatin assembly proteins, and cytoskeletal proteins and associated motors. Smooth muscle cells were transfected and DNA pull-downs were performed at later times and several key proteins were evaluated by Western blot. Several import factors found in the *in vitro* assay also bound *in vivo* to both the SV40 DTS plasmid as well as an SMGA DTS plasmid, but not pBR322. These factors included importin β1, importin 7, and Ran. Since our previous data using the permeabilized cell model pointed to a role for importin β1 in plasmid nuclear localization [66], we depleted importin β1 or importin 7 from the cells using siRNA. Depletion of importin β1 abolished nuclear import of plasmids carrying either the SV40 DTS or the SMGA DTS following cytoplasmic microinjection. By contrast, depletion of importin 7 (>90% depletion by Western) had no discernable effect on the nuclear import of either plasmid in smooth muscle cells, suggesting that importin 7 does not play an active role in plasmid nuclear localization, at least in smooth muscle cells.

Ran was also identified to be interacting with plasmid DNA in another *in vitro* study using a similar affinity chromatography approach but using extracts from HeLa cells [103]. Other proteins shown to bind to a DTS-containing supercoiled plasmid included several chaperones, transcription factors, cytoskeletal proteins, histone H2B and NM23-H2, a transcription factor and metastasis suppressor [103]. When these proteins were depleted from the HeLa cytoplasmic extracts that support plasmid nuclear import in permeabilized cells, the depleted extracts no longer promoted plasmid nuclear import. Further, when purified NM23-H2 and Hsc70 (a chaperone shown to bind to the plasmid during affinity chromatography) were added back to the depleted HeLa cytoplasmic extract, plasmid nuclear import was completely restored. Whether NM23-H2 mediates its effects on DNA nuclear import through its DNA-binding activity or through its kinase activity remains to be determined, but it appears to have clear effects on DNA uptake.

Other studies have also identified importin family members as playing a role in DNA nuclear uptake. Several studies looking at the nuclear targetting of HIV reverse-transcribed DNA complexes pointed to a role for importin 7 [104,105]. When importin 7 was knocked down in HeLa cells using shRNA, transfection of a linearized HIV-1 plasmid was reduced five-fold compared with control cells [105]. Based on this finding, recombinant importin 7 was tested in a permeabilized cell assay and shown to support the nuclear import of fluorescently labeled plasmids in the presence of energy and Ran [106]. Surprisingly, in this study, importin β1 also stimulated plasmid nuclear import, although the authors indicated that the increase was not significantly increased over that seen with Ran and energy alone. When importin 7 was depleted from cells using shRNA (~90% knockdown) and the cells were transfected with an SV40 enhancer containing plasmid complexed with poly-L-lysine, nuclear levels of DNA were decreased by ~60% compared with control. These results are in contrast with those seen in our studies where knockdown of importin 7 by the same degree in either smooth muscle cells or A549 cells caused no change in nuclear accumulation of labeled plasmids [2,3]. It is possible that complexation with poly-L-lysine and transfection as opposed to direct injection into the cytoplasm could account for these differences. However, it has been shown that histone H1 is imported into the nucleus by interacting with a heterodimeric importin β1/importin 7 complex and it is possible that both importin 7 and β1 could play roles in the import of H1-bound DNA complexes [107].

Alternatively, it is also possible that the fluorescent labeling methods could have an effect on the nuclear import of the DNA. In our studies, fluorophore-labeled PNAs (PNA Bio Inc, Newbury Park, California, USA) were bound to plasmids in a triplex structure that is stable in cells and serum for several days [66,108–110], whereas in the study by Dhanoya and colleagues [106], plasmids were labeled with the intercalating YOYO-1 dye. While YOYO-1 binds to DNA with high affinity and is extremely stable for electrophoresis at low salt concentrations, at physiological salt concentrations, dissociation of YOYO-1 can occur within seconds to minutes [111,112]. It has been found that labeling methods for plasmids can have profound effects on the DNA in terms of transcriptional activity or subcellular localization. This is most notable for dyes that covalently attach to the DNA at random sites, especially when labeled at a high fluorophore to DNA ratio. Indeed, when plasmids were labeled at high labeling ratios with one of several different commercially available photoactivatable or chemical DNA labeling kits (Label IT or Fast Tag), the ability of the plasmids to enter the nucleus was destroyed as was their transcriptional activity [61,113]. In either case, when bulky adducts are added randomly to the DNA, it is not surprising that the ability of proteins to bind to the DNA can be reduced, if the adducts are coincident with the binding sites.

Transportin (also known as importin β2) is another importin family member that mediates the nuclear import of the M9 class of NLS found on the hnRNP A1 protein [114,115]. Transportin also has been shown *in vitro* to mediate the nuclear import of H2A, H2B, H3, and H4 histones [116]. Nuclei reconstituted *in vitro* from *Xenopus* egg extracts show high fidelity NLS-mediated nuclear import in a defined system that can easily be manipulated [117]. This system also has been used to study the nuclear import of 400–1500 bp DNA fragments [118]. When reconstituted from extracts depleted of transportin, the nuclei failed to import DNA, while reconstitution of nuclei from extracts devoid of importin β1 had little defect in this regard. A similar need for transportin was also shown in digitonin-permeabilized HeLa cells [118]. These authors showed that the nuclear import mediated by transportin was through the binding of core histones to the DNA. Interestingly, transportin and importin 4 were also identified by MS to bind to nuclear import competent plasmids in pull-down experiments in electroporated cells, although the specific roles of these proteins in nuclear import was not further investigated [2]. Since all the core histones were also shown to complex with the DNA in these transfected cells at early times after electroporation [2], it is possible that the same mechanisms may apply to intact plasmids.

## Conclusion

It is vital that we understand how plasmids move inside the cell in order to develop novel ways to enhance trafficking and improve gene delivery and expression. Although the intracellular trafficking of exogenous DNA may not be a normal event in the cell, mechanisms do exist for its transport. Some of these have evolved, as viruses and other pathogens have perfected ways to invade the host, while others appear to be fortuitous piracy, as in the case of the SV40 DTS, which binds to transcription factors and other proteins destined for the nucleus.

By characterizing and understanding the mechanisms of the cytoplasmic trafficking and nuclear import of plasmids, we can overcome our relative inability to target substantial amounts of DNA to the nucleus and increase transfection efficiency, and ultimately gene therapy.

## Abbreviations

CMV: cytomegalovirus
CREB: cAMP response element binding protein
DTS: DNA nuclear targetting sequence
HDAC6: histone deacetylase 6
HRE: hypoxia-responsive element
LTR: long terminal repeat
MAP: microtubule-associated protein
NES: nuclear export signal
NLS: nuclear localization signal
NPC: nuclear pore complex
PNA: peptide nucleic acid
SMGA: smooth muscle γ actin
SPC: surfactant protein C
SRF: serum response factor
